# Awareness of age-related change in the context of major life events

**DOI:** 10.3389/fpsyt.2022.954048

**Published:** 2022-10-28

**Authors:** Fiona S. Rupprecht, Serena Sabatini, Manfred Diehl, Denis Gerstorf, Roman Kaspar, Oliver K. Schilling, Hans-Werner Wahl

**Affiliations:** ^1^Department of Developmental and Educational Psychology, University of Vienna, Vienna, Austria; ^2^Università della Svizzera Italiana, Faculty of Biomedical Sciences, Lugano, Switzerland; ^3^Department of Human Development and Family Studies, Colorado State University, Fort Collins, CO, United States; ^4^Institute for Psychology, Humboldt University of Berlin, Berlin, Germany; ^5^Cologne Center for Ethics, Rights, Economics, and Social Sciences of Health, Cologne, Germany; ^6^Psychological Institute, Ruprecht-Karls University Heidelberg, Heidelberg, Germany

**Keywords:** subjective aging, family events, health events, lifespan development, developmental change, developmental gains, developmental losses

## Abstract

Although gains and losses are an integral part of human development, the experience of change and readjustment that often comes with major life events may be particularly influential for an individual's subjective aging experience and awareness of age-related change (AARC). Thus, this study focused on the role of life events in the domains of family and health for an individual's awareness of age-related gains and losses. Specifically, we differentiated between the experience of specific life events (e.g., entering a new romantic relationship; hospital stay) and the cumulative experience of multiple life events. Furthermore, we differentiated between life events experienced at an expected time in life and life events experienced relatively early or relatively late compared to established social norms. Data came from the Innovation Sample of the German Socio-Economic Panel (SOEP-IS) and consisted of 1,612 participants aged 16 to 93 years (*M* = 54.1; *SD* = 18.2). Life events were assessed annually and retrospectively for the last 2 years. Propensity score matching provided evidence for an association of specific family life events and a higher awareness of age-related gains, as well as specific health life events and a higher awareness of age-related losses. Results furthermore indicated that the cumulative experience of family life events was associated with a higher awareness of age-related gains. Conversely, the cumulative experience of health events was associated with higher awareness of both age-related losses and age-related gains. Moreover, it was not only life events happening at an expected age, but also those happening relatively early and particularly those happening late in life, which were associated with AARC. In summary, life events and the change they may bring seem to be reflected in individuals' awareness of age-related losses and awareness of age-related gains.

## Awareness of age-related change in the context of major life events

Both gains and losses are seen as a natural part of human development and aging (1). The awareness of age-related gains and losses (i.e., AARC-gains and AARC-losses), however, differs between individuals and has been linked to various developmental outcomes, such as psychological wellbeing, mental health, physical health, and even survival—with AARC-losses exhibiting a mostly negative association with development outcomes, and AARC-gains showing oftentimes a small but positive association (2–4). Qualitative research has pointed toward critical life events, such as new medical diagnoses or entering retirement, as potential contributors to perceiving AARC-gains or AARC-losses (5). Life events are seen as specific transitions and discontinuities in an individual's life course and elicit a general awareness of change and readjustment (6, 7). As many life events in adulthood are to some extent age-graded, it seems plausible that they may trigger a greater awareness of *age-related* change and, thus, contribute to both AARC-gains and AARC-losses. Therefore, the current work investigates AARC in the context of life events.

### Awareness of age-related change

Subjective aging of which AARC is a central component refers to individuals' reflection, understanding, and identification with their personal aging processes (8, 9). AARC is defined as “all those experiences that make a person aware that [their] behavior, level of performance, or ways of experiencing [their] life have changed as a consequence of having grown older (i.e., increased chronological age)” [(10), p. 340]. In contrast to most other operationalizations of subjective aging, AARC explicitly acknowledges the coexistence of gains and losses throughout human development (1, 10). This aspect is particularly relevant when studying the context of life events, which can simultaneously come with gains and losses. For example, both negative *and* positive life events are seen as major stressors and challenges for an individual's wellbeing and mental health (7, 11, 12), but can also result in psychological growth (13, 14). Furthermore, AARC is conceptualized as comprising multiple dimensions, including changes in health and physical functioning, cognitive functioning, interpersonal relations, social-cognitive and social-emotional functioning, and lifestyle and engagement, allowing to capture the broad range of adaptation that is often required when dealing with life events (15, 16).

Whereas research has been accumulating on individual difference predictors of AARC (17, 18), research on the effects of context-based predictors of AARC is still very much at the beginning (19). Reasoning behind the construct of AARC itself (20) as well as other indicators of subjective aging (21, 22) point to the need to contextualize AARC and thus, to better understand how “diverse […] phenomena, events, and forces that exist outside of the developing individual” [(23), p. 84] shape the subjective experience of aging and change. Life events are usually either elicited by contexts or heavily involve (changing) contexts and are thus crucial when aiming for a contextualization of AARC. In the following, we first discuss the potential role of specific life events in the domains of family and health for AARC. Subsequently, we focus on the role of accumulating life events for AARC. Lastly, we discuss the role of timing and age-normativity of life events when it comes to their relation to AARC.

### Specific life events and AARC

According to life script theory, specific major life events, such as the birth of a child, the death of a parent, or the diagnosis of a critical illness, are used as developmental markers and help to structure the human life course (24). Within cultures, there is often much consensus about the order and timing of life events; that is, at what age life events are considered normative (25, 26). This age-normativity of a considerable portion of major life events is closely linked to life anticipation as well as the achievement of important life goals. At the same time, such life events infuse and structure autobiographical memories and meaning-making of a person's individual development (24, 26). Because AARC is essentially one possible way to reflect and make meaning of a person's own development and strongly symbolizes that lifetime is progressing, it stands to reason that specific, age-normative life events may feature strongly in an individual's awareness of age-related gains and losses.

Prior research regarding life events and subjective aging has mostly focused on such age-normative life events and their relation to the *felt age* of individuals. Within this line of research, it has been argued that life events may serve as markers of aging processes and could thus affect how old people feel (22). Whereas specific life events in the family domain (e.g., marriage or death of one's spouse) have been found to be unrelated to (changes in) people's felt age (27–29), specific life events in the health domain (e.g., incident of a serious disease) were indeed linked to increases in felt age over time (27). We therefore distinguish between life events in the *family domain*, which involve individuals' close others and comprise experiences of both gains and losses, and life events in the *health domain*, which often involve losses and may be more readily attributed to the aging process (30). The existing lifespan literature argues that the social/family and health domain are among the most important drivers of development across the adult lifespan (31, 32). Consequently, we first investigate the association of specific life events in the health and family domain with AARC. This should allow us to examine whether there are life events (e.g., birth of a child for young adults, new medical diagnoses for older adults) that may serve as markers of aging and thus prompt AARC-gains or AARC-losses.

### Accumulated life events and AARC

Irrespective of their potential age association, major life events often constitute transitions and discontinuities in a person's life course and everyday life routines and require instrumental, behavioral, cognitive, and emotional adaptation (7, 16). They often come with immediate changes to social networks, health, functioning, finances, and daily life, as well as new social roles, tasks, demands, and requirements and can, eventually, lead to changes in personality and the self (15, 16). Life events can thus be seen as drivers of change and challenges for psychological adaptation and development. When life events accumulate, their consequences tend to accumulate as well (33) and may profoundly affect an individual's level of adjustment and quality of life. How successfully or unsuccessfully individuals deal with this challenge of accumulated change, the associated stress, increasing constraints and a lack of control (34, 35), may generally foster a strong awareness of age-related change. For example, individuals could become aware of their age-related gains in socio-emotional functioning that now allow them to cope with the change better than they would have been able to at a younger age. It also stands to reason that life events themselves may not be linked to age, but the consequences for one's individual development, for example in health or interpersonal relations, may still be attributed to age.

When taking on this perspective of accumulated life events as a challenge for development, it should not be the influence of specific (age-related) life events, but rather their accumulation: The higher the number of life events an individual has experienced within a specific time period, the more pronounced the experience of change and the required adaptation and—potentially—the increase of AARC-gains and AARC-losses. For testing this hypothesis, we utilized two count variables for life events, one for the family domain and the other for the health domain, as a measure of accumulated life events.

### Timing of life events and AARC

Whereas our investigation of single life events in relation to AARC only allows for a qualitative comparison of more or less age-normative life events (e.g., birth of a child as a highly age-normative life event for young adulthood), the analysis of accumulated life events comes with enough statistical power for a more fine-grained investigation of the timing of life events and the association of timing with AARC. One assumption would be that age-corresponding life events (i.e., life events experienced at an age, at which they are expected or normative) may feature more strongly in an individual's AARC because changes elicited by the age-corresponding life events could easily be interpreted as age-related. The contrasting assumption would, however, be that the experience of life events at a non-normative or uncommon age (either relatively early or relatively late) could make a person's diverging and non-normative aging process particularly salient and contribute to a strong individual AARC. The uncommon or non-normative timing makes life events and the associated changes less predictable and less amenable to anticipatory coping and personal control. Moreover, individuals who experience life events at a non-normative time may have less social support available to them and may be more prone to engage in unfavorable social comparisons (36–38). All of these aspects may affect the association of life events with AARC. In a third step, we thus compared the (cumulative) experience of life events happening relatively (or non-normatively) early, life events happening relatively (or non-normatively) late, and life events happening at an expected (or normative) age in respect to their relation to AARC.^1^ This allowed us to investigate whether the timing of (cumulative) life events plays a role for the association between life events and AARC.

### Current study

The current study focused on awareness of age-related change in the context of life events. We investigated (a) whether single and specific life events were related to AARC, (b) whether the cumulative experience of life events was related to AARC, and (c) the role of timing of these life events. For all research questions, we applied a lifespan perspective on AARC and included individuals in early, middle, and late adulthood in the analyses. We do so because change in form of both gains and losses is seen as a ubiquitous phenomenon throughout the lifespan (1) and reflections upon and interpretation of aging should happen across adult development. In addition, given our argument on the role of timing of life events, a research approach including AARC in early adulthood is of particular importance.

Regarding the first research question, we started by investigating single life events and their role for AARC by propensity score matching individuals who had experienced a respective life event with highly similar individuals who had not experienced this event. We expected traditional age-related life events, such as the birth of a child, to be particularly strongly related to AARC. We investigated the second research question by using count scores for life events and assuming that a higher number of life events was associated with accumulated change and could thus be related to a higher awareness of age-related change. For the third research question, we were interested whether the point in life at which life events occurred was relevant for their association with AARC. There are arguments for both a particularly strong association between *age-corresponding* life events and AARC as well as a particularly strong association between relatively early or late life events and AARC. Therefore, we considered this an exploratory research question, which we also addressed using count variables for *age-corresponding life events, early life events*, and *late life events*.

Regarding the choice of life events, we decided to focus on *family life events* and *health life events*. Family life events encompass events concerning romantic relationships, children, and death, and allow for a strongly contextual perspective on AARC as all those events involve persons other than the participating individuals themselves. Health life events focus on new mental and physical health diagnoses, as well as hospital stays. Due to the often strong association between health and AARC-losses (4), we expected health life events to be more strongly related to AARC-losses. Overall, we however acknowledge that life events may feature gains and losses at the same time and thus expected them to be related with potential increases in both, AARC-losses and AARC-gains. For time distance, we focused on life events that had occurred within the 2 years before the assessment of AARC. We chose this time distance because dynamic adaptation to most life events usually takes place within 2 years after the occurrence of the event (39). All analyses controlled for the influence of age and gender, which are oftentimes related to the actual occurrence of life events. To account for resources of the participants in dealing with the life events, we furthermore controlled for their education, their number of close friends (as a social resource variable), and their previous health state, that is, the health state an individual had before the health life events occurred.

## Method

### Sample and procedure

Data came from the Innovation Sample of the German Socio-Economic Panel Study (SOEP-IS). The SOEP study is a multidisciplinary and longitudinal survey covering ~11,000 German households. SOEP-IS is one of its extensions and applies innovative research questions and instruments not covered in the core survey to smaller subsamples (40). Participants in two of these subsamples answered the items on awareness of age-related change and, thus, formed the sample for the present study. The participants were 1,612 adults from 1,011 German households aged 16 to 93 years (*M* = 54.1 years; *SD* = 18.2 years), and 53% of them were women. Among the participants, 53% were married, 2% were married but lived in separation, 24% were single and never married, 12% were divorced and 9% were widowed. The sample reflected diverse educational backgrounds with 1% still being in school, 6% either not having completed school or only having completed general elementary school, 4% having completed intermediate school, 14% having a general or vocational maturity certificate, 24% having a basic vocational and 30% having intermediate vocational qualification, 5% having lower tertiary and 14% having higher tertiary education. Monthly household net incomes ranged from 129€ to 21,250€, *Md* = 2,700€, *M* = 3,043€, *SD* = 1,718€. 10% of the participants had direct migration background, 9% had indirect migration background (i.e., participants were born in Germany, but have at least one parent who immigrated to Germany).

### Measures

#### Awareness of age-related gains and losses

Awareness of age-related gains and losses was assessed with the 10-item short form of the AARC questionnaire, which was specifically developed for large-scale studies (41). Items start with the phrase “With my increasing age, I realize that…” and conclude with a gain- or loss-specific statement reflecting one of the five domains *health and physical functioning, cognitive functioning, interpersonal relations, social-cognitive and social-emotional functioning*, and *lifestyle and engagement*. For example, for the domain of social-cognitive and social-emotional functioning the two statements are “…I have a better sense of what is important to me” and “…I find it harder to motivate myself”. Items were answered on a scale ranging from 1 (*not at all*) to 5 (*very much*). The AARC-gains and AARC-losses subscales were computed by averaging the respective five items. Awareness of age-related gains amounted to *M* = 3.30, *SD* = 0.74, and had a reliability of Cronbach's α = 0.66. Awareness of age-related losses amounted to *M* = 2.09, *SD* = 0.80, and achieved good reliability with Cronbach's α = 0.81. The two subscales were positively related to each other, *r* = 0.28, *p* < 0.001.

#### Life events in the family domain

Participants were asked whether the following changes occurred in their life since the beginning of 2016, or the beginning of 2017, respectively (measurement points conducted in 2017 and 2018 were used): New romantic partnership, moving in with partner, new marriage, birth of child, child moving in, child moving out, separation from partner, divorce, death of partner, death of father, death of mother, death of child, death of (another) household member, (other) change in household composition. Due to available assessment format, there is only information whether a life event occurred (1) or not occurred (0) at least once during the last 2 years. Multiple occurrences of the same life event could not be operationalized. Life events in the family domain were summed up to the count variable *family life events*, which ranged from 0 to 5, *Md* = 0, *M* = 0.41, *SD* = 0.69.

In a next step, we split the count variable *family life events* into three distinct count variables, one for life events happening non-normatively early (i.e., *early family life events*), one for life events happening within a normative age range (i.e., *age-corresponding family life events*), and one for life events happening non-normatively late (i.e., *late family life events*). Hereby, the 25% and 75% age quartiles were determined across all participants who had experienced a specific life event within the last 2 years. If a participant had experienced a specific life event themselves and their age lay below the 25% quartile it was counted as an *early* life event, if their age lay above the 75% quartile it was counted as a *late* life event, and if it lay between the 25% and the 75% quartile (i.e., in the interquartile range) it was counted as an *age-corresponding* life event.

#### Life events in the health domain

Participants were asked whether a physician had diagnosed the following diseases within the last year, both in 2017 and in 2018: Sleep disorder, diabetes, asthma, cardiac disease, cancer, apoplectic stroke, migraine, depressive disorder, high blood pressure, dementia, joint disorder, chronic back pain, or any other disease. In order to unambiguously identify new medical diagnoses, we only counted diagnoses that had not yet been reported by the individual in the years of 2012 to 2015. Additionally, participants were asked whether they had been admitted to a hospital at least once in the two previous years. Multiple occurrences of the same life event were again not assessed. Life events in the health domain were summed up to the count variable *health life events*, which ranged from 0 to 5, *Md* = 0, *M* = 0.59, *SD* = 0.85. In a next step, the three variables *early health events, age-corresponding health events*, and *late health events* were formed as described for family life events.

#### Covariates

As covariates for the regression analyses and as predicting variables in the propensity score matching, we used age, sex, education, number of close friends and prior count of medical diagnoses. Age, sex, and education were used to account for demographic characteristics of the individual. Age was coded as years since birth, sex was coded as 0 for *women* and 1 for *men*, and education was coded using the Comparative Analysis of Social Mobility in Industrial Nations (CASMIN) indicator, which ranges from *in school* (0) to *higher tertiary education* (9). The number of close friends reflects an important social resource for dealing with life events in both the health and the family domain (42). It was assessed directly with the question “How many close friends would you say you have” and subsequently reduced to a maximum number of 35, which reflects the assumed limit of close social relationships an individual can have (43). The prior count of diagnoses refers to the health state of an individual in the years between 2012 and 2015—and thus, before the *health life events* and *family life events* occurred and is based on the same list of diagnoses as the variable of *health life events*.

### Analyses

To test the role of single life events, we first identified the individuals who had experienced the specific life event within the last 2 years. We then matched these individuals who had experienced the life event (experiential group; Exp) to highly similar individuals who had not experienced the life event (control group; Con). This was done by propensity score matching, wherein each individual was assigned a probability of having experienced the respective life event using logistic regression analysis and age, sex, education, number of close friends, and prior count of diagnoses as predictors. Individuals from the experiential group were then matched to their closest neighbor from the control group. Subsequently, the individuals in the experiential and the control group were compared in their AARC-gains and AARC-losses scores using paired t-tests. As the number of individuals who had experienced a specific life event was often small, we performed power analyses beforehand to estimate the size of effects we would be able to find. Power analyses indicated that 33 pairs of individuals would be enough to detect moderate-sized effects (*d* = 0.50). Detecting weaker effects (*d* = 0.40, *d* = 0.30, *d* = 0.20) would require larger group sizes, respectively (*n* = 51, *n* = 89, *n* = 198). We thus only conducted the *t*-tests for life events with an experiential group of *n* ≥ 33.

To test the role of accumulated life events, we performed linear regression models with AARC-gains and AARC-losses as the respective outcome variables. In a first analysis, the two general count variables *family life events* and *health life events* served as predictors. In a second analysis, we differentiated between life events experienced at a normative age and non-normatively early/late by focusing on *early family life events, age-corresponding family life events, late family life events, early health life events, age-corresponding health life events*, and *late health life events* as predictors. Age, sex, education, number of close friends, and prior count of diagnoses served as covariates in all analyses. R version 4.1.1 was used for analyses (44).

## Results

Descriptive statistics and bivariate correlations of the study variables are shown in Table 1. At the bivariate level, the higher the number of health events (general, age-corresponding, and late), the older the age, the larger the number of close friends and the more prior diagnoses, the higher was the reported AARC-gains. Also, men reported slightly higher AARC-gains than women. The higher the number of family life events (general, early, and age-corresponding), the number of health events (general, age-corresponding, and late), the age, and the count of prior diagnoses, but the lower the education and the number of close friends, the higher was the reported AARC-losses. The experience of family life events was associated with the experience of health life events (more early health life events, but less late health life events), a younger age, a higher education, and a lower count of prior diagnoses. The experience of health life events was furthermore associated with an older age, lower education, and a higher count of prior diagnoses.

**Table 1:** Descriptives and bivariate correlations of the study variables (*N* = 1,612).

**Variable**	**M(SD)**	**1**.	**2**.	**3**.	**4**.	**5**.	**6**.	**7**.	**8**.	**9**.	**10**.	**11**.	**12**.	**13**.	**14**.
1. AARC-gains	3.30 (0.74)														
2. AARC-losses	2.09 (0.80)	**0.28**													
3. Family life events	0.41 (0.69)	0.04	**−0.14**												
4. Early family life events	0.10 (0.35)	−0.01	**−0.14**	**0.48**											
5. Age-corresponding family life events	0.21 (0.50)	0.02	**−0.10**	**0.74**	0.01										
6. Late family life events	0.10 (0.33)	0.05	0.00	**0.47**	**−0.07**	0.04									
7. Health life events	0.59 (0.85)	**0.12**	**0.26**	−0.03	−0.03	−0.03	0.02								
8. Early health life events	0.14 (0.45)	−0.04	−0.05	**0.13**	**0.19**	**0.09**	**−0.06**	**0.44**							
9. Age-corresponding health life events	0.30 (0.62)	**0.09**	**0.15**	−0.05	−0.12	−0.02	**0.06**	**0.68**	**−0.06**						
10. Late health life events	0.14 (0.48)	**0.14**	**0.31**	**−0.12**	**−0.08**	**−0.12**	0.02	**0.49**	**−0.09**	−0.03					
11. Age	54.13 (18.18)	**0.18**	**0.40**	**−0.30**	**−0.39**	**−0.23**	**0.13**	**0.16**	**−0.35**	**0.17**	**0.40**				
12. Gender	0.47 (0.50)	−0.05	0.04	−0.03	−0.03	−0.04	0.03	−0.01	**−0.05**	0.00	0.02	0.00			
13. Education	5.14 (2.29)	−0.04	**−0.17**	**0.08**	0.00	**0.11**	0.00	**−0.11**	−0.05	−0.03	**−0.11**	**−0.11**	0.02		
14. Close friends	4.42 (3.69)	**0.06**	**−0.07**	0.03	−0.02	0.03	0.04	−0.02	−0.03	−0.01	0.00	0.04	0.00	**0.10**	
15. Prior diagnoses	0.91 (1.19)	**0.10**	**0.31**	**−0.09**	**−0.10**	**−0.10**	**0.07**	**0.12**	**−0.10**	**0.09**	**0.19**	**0.39**	−0.04	**−0.14**	−0.04

Significant (p < 0.05) bivariate correlations are printed bold.

### The relation between specific life events and AARC

Associations between the experience of specific *family* life events and awareness of age-related change are shown in Table 2. Only the start of a new romantic relationship as well as the separation from one's partner were significantly related to a higher awareness of age-related gains. No specific family life event was related to AARC-losses. Associations between the single *health* life events and awareness of age-related change are shown in Table 3. The new diagnosis with a sleep disorder or a cardiac disease were associated with a stronger awareness of age-related losses. Similarly, individuals who had a hospital stay within the last 2 years, reported a slightly elevated awareness of age-related losses. No specific health life event was related to AARC-gains. To indicate the age characteristics of the individuals who had experienced a specific life event, their age range, age mean, and standard deviation are provided in Tables 2, 3.

**Table 2:** Investigating the impact of specific life events in the family domain using propensity score matching.

		**Age**	**AARC-gains**				**AARC-losses**			
**Life Event**	***n***	**Range, *M* (*SD*)**	***M* (*SD*)_Exp_**	***M* (*SD*)_Con_**	***t* (df)**	***d***	***M* (*SD*)_Exp_**	***M* (*SD*)_Con_**	***t* (df)**	***d***
New romantic relationship	98	17–78, 35.54 (14.57)	**3.47 (0.62)**	**3.04 (0.82)**	**3.89 (94)**	**0.40**	1.81 (0.71)	1.73 (0.65)	0.88 (94)	0.09
Moving in with partner	63	20–65, 35.60 (11.23)	3.35 (0.52)	3.17 (0.76)	1.63 (61)	0.21	1.72 (0.60)	1.65 (0.69)	0.61 (61)	0.08
New marriage	41	21–65, 35.29 (11.00)	3.38 (0.44)	3.33 (0.84)	0.31 (38)	0.05	1.74 (0.64)	1.68 (0.66)	0.40 (38)	0.06
Birth of child	46	20–44, 33.57 (5.01)	2.97 (0.66)	2.98 (0.84)	**–**0.11 (45)	**–**0.02	1.64 (0.59)	1.72 (0.69)	**–**0.67 (45)	**–**0.10
Child moving in	61	21–63, 40.66 (10.07)	3.21 (0.62)	3.05 (0.80)	1.14 (60)	0.15	1.77 (0.65)	1.81 (0.57)	**–**0.29 (60)	**–**0.04
Child moving out	80	29–87, 54.01 (8.61)	3.40 (0.64)	3.28 (0.82)	1.08 (79)	0.12	2.05 (0.65)	2.15 (0.80)	**–**0.82 (79)	**–**0.09
Separation from partner	41	18–88, 40.07 (14.88)	**3.54 (0.59)**	**3.14 (0.66)**	**2.91 (40)**	**0.45**	1.83 (0.74)	2.03 (0.84)	**–**1.16 (40)	**–**0.18
Divorce	6	29–53, 39.33 (7.94)								
Death of partner	18	52–89, 70.44 (10.98)								
Death of father	37	27–68, 52.89 (11.79)	3.34 (0.72)	3.52 (0.83)	**–**1.25 (36)	**–**0.21	2.04 (0.80)	2.31 (0.98)	**–**1.26 (36)	**–**0.21
Death of mother	50	24–81, 56.76 (12.89)	3.42 (0.70)	3.38 (0.75)	0.23 (49)	0.03	2.11 (0.86)	2.22 (0.76)	**–**0.71 (49)	**–**0.10
Death of child	7	40–85, 62.57 (17.92)								
Death of household member^a^	16	20–80, 48.50 (17.37)								
Change in household composition^a^	102	17–90, 51.37 (20.25)	3.30 (0.78)	3.40 (0.72)	**–**0.99 (98)	**–**0.10	1.93 (0.83)	2.06 (0.83)	**–**1.09 (98)	**–**0.11

n indicates the number of individuals who experienced the respective life event within the last 2 years. The age characteristics are given for the individuals in the Exp group. In case n ≥ 33, the individuals who experienced the life event (Exp) are matched with a control group of individuals who did not experience the life event (Con) using propensity score matching. The groups are compared using paired t-tests. Significant comparisons (p < 0.05) are printed bold. Effect sizes are given as d.

^a^ The two events only encompass deaths and changes not already targeted within over life events.

**Table 3:** Investigating the impact of specific life events in the health domain using propensity score matching.

		**Age**	**AARC-gains**				**AARC-losses**			
**Life Event**	***n***	**Range, *M* (*SD*)**	***M* (*SD*)_Exp_**	***M* (*SD*)_Con_**	***t* (df)**	***d***	***M* (*SD*)_Exp_**	***M* (*SD*)_Con_**	***t* (df)**	***d***
Diagnosis of sleep disorder	56	18–86, 53.70 (18.27)	3.23 (0.68)	3.17 (0.79)	0.41 (55)	0.05	**2.23 (0.84)**	**1.94 (0.72)**	**2.06 (55)**	**0.28**
Diagnosis of diabetes	21	17–89, 63.95 (17.58)								
Diagnosis of asthma	27	21–86, 61.85 (20.40)								
Diagnosis of cardiac disease	51	22–90, 70.55 (12.28)	3.61 (0.71)	3.58 (0.66)	0.23 (50)	0.03	**2.66 (0.87)**	**2.22 (0.73)**	**3.16 (50)**	**0.44**
Diagnosis of cancer	30	19–86, 65.03 (16.08)								
Diagnosis of apoplectic stroke	10	62–90, 73.20 (10.53)								
Diagnosis of migraine	24	17–86, 42.17 (21.19)								
Diagnosis of high blood pressure	79	20–86, 60.49 (13.37)	3.36 (0.74)	3.25 (0.76)	0.90 (78)	0.10	2.28 (0.89)	2.14 (0.74)	1.22 (78)	0.14
Diagnosis of depressive disorder	38	18–84, 47.26 (20.13)								
Diagnosis of dementia	6	44–83, 70.50 (17.97)								
Diagnosis of joint disorder	85	20–93, 60.56 (16.11)	3.46 (0.66)	3.40 (0.63)	0.62 (84)	0.07	2.38 (0.83)	2.18 (0.86)	1.62 (84)	0.18
Diagnosis of chronic back pain	75	20–87, 59.72 (18.05)	3.40 (0.73)	3.36 (0.79)	0.33 (73)	0.04	2.41 (0.93)	2.13 (0.78)	1.88 (73)	0.22
Diagnosis of other illness	76	17–85, 52.63 (19.73)	3.32 (0.78)	3.38 (0.73)	−0.43 (70)	−0.05	2.30 (0.91)	2.09 (0.73)	1.59 (70)	0.19
Hospital stay	366	17–90, 57.95 (18.35)	3.43 (0.67)	3.35 (0.70)	1.57 (355)	0.08	**2.35 (0.87)**	**2.21 (0.81)**	**2.25 (355)**	**0.12**

n indicates the number of individuals who experienced the respective life event within the last 2 years. The age characteristics are given for the individuals in the Exp group. In case n ≥ 33, the individuals who experienced the life event (Exp) are matched with a control group of individuals who did not experience the life event (Con) using propensity score matching. The groups are compared using paired t-tests. Significant comparisons (p < 0.05) are printed bold. Effect sizes are given as d.

### The relation between cumulative life events and AARC

Results of AARC's prediction by the general count variables are shown in Table 4. Awareness of age-related gains was stronger when individuals had experienced an overall higher number of both family and health life events in the two previous years. Furthermore, AARC-gains was positively associated with an older age, female sex, and a higher number of close friends. After accounting for covariates, the two general count variables for life events accounted for an additional 1.5% of variance in AARC-gains, which indicates a small effect size.

**Table 4:** Investigating the predictive effects of accumulating life events for awareness of age-related change.

	**AARC-gains**		**AARC-losses**	
**Variable**	**B (SE)**	**β**	**B (SE)**	**β**
Intercept	3.24 (0.03)		1.96 (0.03)	
Family life events	0.10 (0.03)	**0.10**	−0.03 (0.02)	−0.02
Health life events	0.07 (0.02)	**0.09**	0.17 (0.02)	**0.18**
Age	0.01 (0.00)	**0.19**	0.01 (0.00)	**0.30**
Sex	−0.08 (0.04)	–**0.05**	0.09 (0.04)	**0.05**
Education	−0.00 (0.01)	−0.01	−0.03 (0.01)	–**0.09**
Close friends	0.01 (0.00)	**0.05**	−0.01 (0.00)	–**0.07**
Prior diagnoses	0.02 (0.02)	0.03	0.11 (0.02)	**0.16**

Unstandardized coefficients are shown with standard errors in parentheses. Significant effects (p < 0.05) are indicated by the standardized coefficients printed in bold. R^2^_adjusted_ is 5.4% of variance for AARC-gains and 23.8% of variance for AARC-losses. Age, education, close friends, and prior diagnoses are mean-centered.

Awareness of age-related losses was stronger when individuals had experienced an overall higher number of health life events in the two previous years. AARC-losses was not associated with the general number of family life events experienced. Instead, AARC-losses was positively associated with an older age, male sex, lower education, a lower number of close friends, and more prior diagnoses. After accounting for covariates, the two general count variables for life events accounted for an additional 2.9% of variance in AARC-losses, which again indicates a small effect size.

### The relation between the timing of life events and AARC

**Figure 1:**
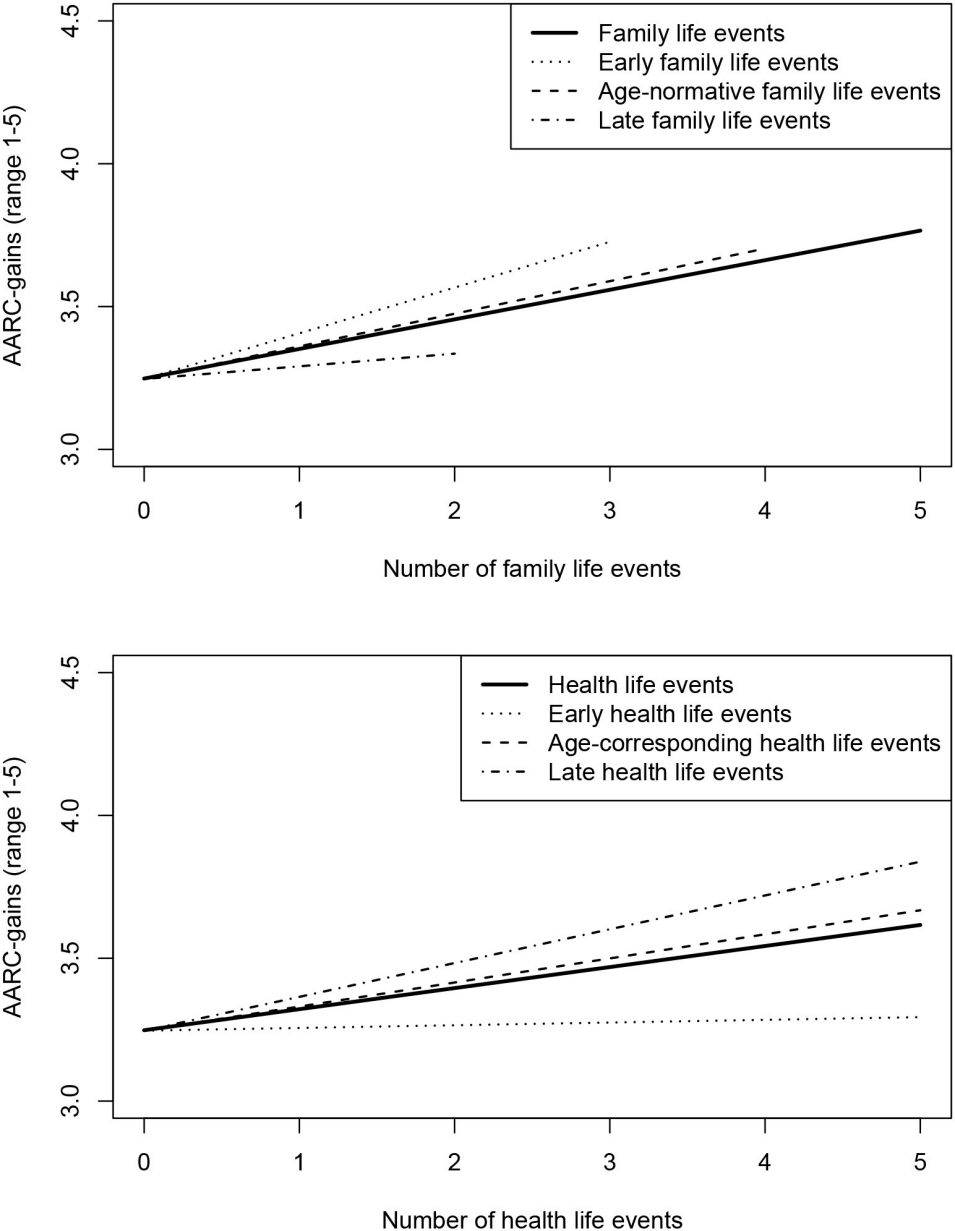
Number of life events and awareness of age-related gains. Regression lines cover the maximum number of life events occurring in the sample.

Table 5 presents the regression analyses with the count variables for (non-normatively) early, age-corresponding, and (non-normatively) late life events instead of the general count variables as predictors of AARC. For AARC-gains, these differentiated count variables did not account for significantly more variance than the general count variables. Still, whereas early and age-corresponding family life events predicted stronger AARC-gains, late family life events did not. In the health domain, age-corresponding and late life events predicted stronger AARC-gains, but early health life events did not. Figure 1 depicts the associations between general, early, age-corresponding, and late life events with AARC-gains.

**Table 5:** Investigating the predictive effects of relatively early, age-corresponding, and relatively late life events for awareness of age-related change.

	**AARC-gains**		**AARC-losses**	
**Variable**	**B (SE)**	**β**	**B (SE)**	**β**
Intercept	3.25 (0.03)		1.96 (0.03)	
Early family life events	0.16 (0.06)	**0.08**	−0.02 (0.06)	−0.01
Age-corresponding family life events	0.11 (0.04)	**0.08**	0.02 (0.04)	0.01
Late family life events	0.04 (0.05)	0.02	−0.13 (0.05)	–**0.05**
Early health life events	0.01 (0.04)	0.01	0.14 (0.04)	**0.08**
Age-corresponding health life events	0.08 (0.03)	**0.07**	0.13 (0.03)	**0.10**
Late health life events	0.12 (0.04)	**0.08**	0.27 (0.04)	**0.16**
Age	0.01 (0.00)	**0.18**	0.01 (0.00)	**0.28**
Sex	−0.08 (0.04)	–**0.05**	0.09 (0.03)	**0.05**
Education	−0.00 (0.01)	−0.02	−0.03 (0.01)	–**0.09**
Close friends	0.01 (0.00)	**0.05**	−0.01 (0.00)	–**0.06**
Prior diagnoses	0.02 (0.02)	0.03	0.11 (0.02)	**0.16**

Unstandardized coefficients are shown with standard errors in parentheses. Significant effects (p < 0.05) are indicated by the standardized coefficients printed in bold. R^2^_adjusted_ is 5.5% of variance for AARC-gains and 24.3% of variance for AARC-losses. Age, education, close friends, and prior diagnoses are mean-centered.

**Figure 2:**
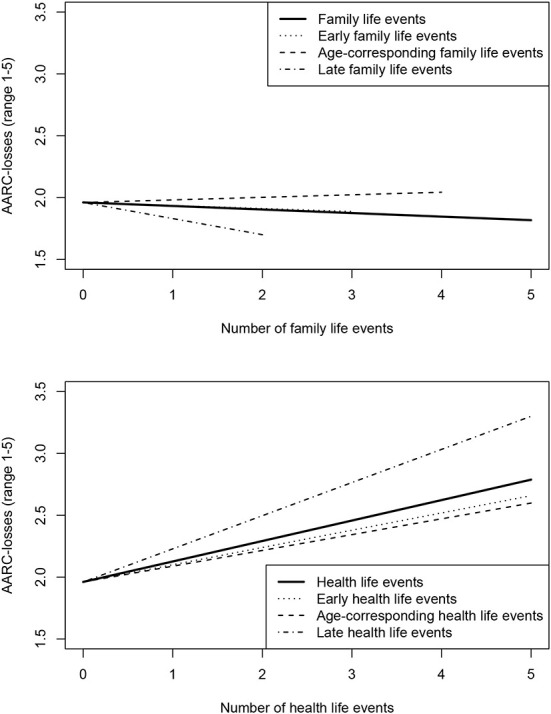
Number of life events and awareness of age-related losses. Regression lines cover the maximum number of life events occurring in the sample.

For AARC-losses, the variance accounted for by the differentiated count variables was slightly higher than the variance accounted for by the general count variables (+0.5%). In the family domain, only late life events were associated with a lower AARC-losses. There was no association between AARC-losses and early and age-corresponding family life events. In the health domain, early, age-corresponding, and late life events were all associated with higher AARC-losses. However, the regression coefficients point toward the strongest association of late health life events with AARC-losses. Figure 2 depicts the associations between general, early, age-corresponding, and late life events with AARC-losses.

## Discussion

This investigation of AARC in the context of life events found a higher number of life events in the family domain to be related to a stronger awareness of age-related gains. A higher number of life events in the health domain was predominantly associated with a stronger awareness of age-related losses but also weakly with a stronger awareness of age-related gains. Associations were particularly evident for life events happening at an age-normative or relatively late time, indicating that the point in life at which a life event was experienced might indeed be relevant. Associations between AARC and single life events were only sporadic but pointed toward a relation between specific family life events and higher AARC-gains and specific health life events and higher AARC-losses. Thus, the distinction between family life events and health life events appeared particularly wellsuited to capture the differential content and relational patterns of AARC-gains and AARC-losses.

The cumulative experience of life events was overall associated with a heightened awareness of age-related change. This indicates that individuals might have interpreted at least part of the change driven by life events as a change also driven by their increasing age. Importantly, this change was not only in the form of losses, but also in the form of gains, hinting toward the growth that can follow even from highly stressful life events (13). Aside from this cumulative effect, we found few single life events that were significantly associated with AARC, that is, *new romantic relationship* and *separation from partner* in the family domain, and *diagnosis of sleep disorder, diagnosis of cardiac disease*, and *hospital stay* in the health domain. Apart from *new romantic relationship*, which is a developmental task traditionally assigned to young adulthood (46), none of the life events seem particularly normative for a certain age group. This finding provides only limited evidence for the role of specific, age-corresponding life events as markers of aging and AARC. This is mostly in line with prior empirical research on single life events outside the health domain which were also unrelated to felt age as a different indicator of subjective aging (27–29). In contrast, the cumulative experience of age-corresponding life events was indeed associated with AARC—though life events happening relatively late in life also appeared of relevance.

### Awareness of age-related change and family life events

The accumulation of life events in the family domain was found to be positively related to AARC-gains. Individuals who experienced (multiple) transitions within their closest relationships and their families seemed to perceive growth and to associate this growth with their own aging. Many life events in the family domain follow from long-term investments (e.g., marriage or child moving out). Experiencing these life events and witnessing the results of such long-term investments may make individuals particularly aware of the gains in their development. This finding highlights the importance to contextualize AARC and to account for individuals' experiences in their families and social networks, which can be one source of positive aging experiences. When differentiating between age-corresponding, relatively early, and relatively late family life events, the two former event types were associated with higher AARC-gains, whereas the latter type was associated with lower AARC-losses. In general, family life events seemed to have positive influences overall, but the exact influences may depend on the point in life at which they are experienced. With regard to specific family life events, only the beginning and ending of a romantic relationship were directly associated with a higher awareness of age-related gains. Changes in the relationship status may come with new experiences, the opportunity to gain new self-knowledge, a heightened appreciation of existing social ties, and overall psychological growth (47) and may thereby raise the awareness of age-related gains.

Somewhat unexpectedly, the negative family life events, which were mainly deaths in the immediate family, were unrelated to both AARC-gains and AARC-losses. However, important moderating aspects, such as the emotional closeness to the person who had died, as well as the circumstances of death could not be examined as this information was not available. Also, there were only very few individuals who reported the death of their spouse and information on the death of other same-aged individuals, such as friends, colleagues, or siblings, was not available. The death of a peer may affect AARC in different and more pronounced ways than for example the death of a parent.

### Awareness of age-related change and health life events

Accumulated life events in the health domain were associated with higher AARC-losses and higher AARC-gains. Regarding the former association, (multiple) diagnoses and health events can signal a deterioration of health, can come with a loss of functioning and independence and may raise an individual's awareness of age-related losses. The close association between health (declines) and a more negative subjective aging experience has been documented in several prior studies (4, 45, 48). The association between health life events and AARC-losses appeared regardless of when they happened—though it appeared strongest for health life events happening particularly late considering the age distribution of the sample.

Regarding single health life events, a new diagnosis with a sleep disorder was related to heightened AARC-losses. This finding is congruent with a recent study linking poor self-reported sleep to higher AARC-losses (49). It also seems important to highlight the weak, though significant impact a hospital stay seemed to have on AARC-losses. Hospital stays are often acute and unexpected and may, thus, be associated with a feeling of vulnerability and lack of controllability. Overall, results suggest that a close monitoring of the aging experience of individuals facing major transitions in the health domain (i.e., multiple diagnoses, hospital stays) could be beneficial, so that losses do not become overwhelming and negatively influence other aspects of life, such as psychological wellbeing (2, 50).

In this vein, the present results offer one potential way of coping with negative life events in the health domain, that is, a parallel awareness of age-related gains. Results indicated that the positive association of health life events with both AARC-losses and AARC-gains could only be found for age-corresponding or late, but not for early life events. Nevertheless, this finding highlights that awareness of growth can happen in the face of health declines and other adversities (13, 14) and that such perceived growth could be explicitly encouraged and supported.

### Limitations and outlook for future research

A first limitation of our research concerns the operationalization of AARC and life events. To our knowledge, the SOEP-IS 2018 provided the first opportunity to assess and investigate AARC in early adulthood. We acknowledge that the assessment of AARC in early adulthood needs more research in the future. However, unpublished psychometric analyses are supporting measurement invariance of the AARC short version across the full adult lifespan (Kaspar et al., in preparation). Life events within the family domain were not necessarily representative for old age and particularly positive life events normative for old age (e.g., birth of a grandchild) were missing. Overall, the distinction between family and health life events proved meaningful in regard to their specific relations to AARC-gains vs. AARC-losses. However, life events in other domains (e.g., work, leisure) could similarly provide distinct influences on AARC and should be investigated in future research. In the framework of SOEP-IS 2018, such other life events were either not assessed or were only applicable for subsamples with fairly limited age ranges (e.g., graduation or retirement). Additionally, the non-occurrence of certain life events (e.g., not receiving medical diagnoses as an older adult or not becoming a parent as a young adult) or the multiple occurrence of the same life event (e.g., new romantic relationship) within 2 years may also be impactful for AARC but could not be examined within this study.

A second limitation concerns the fact that only cross-sectional data were available for AARC. Though information on other variables and life events was available from the years before, AARC was only assessed at a single occasion in 2018. Thus, actual increases and decreases in AARC as a consequence of the experience of life events could not be examined. Relations in the opposing direction, that is AARC as a predicting variable of single life events (e.g., high AARC-gains as a predictor of starting a new romantic relationship; high AARC-losses as a predictor for new medical diagnoses) could also not be ruled out. Future research should concentrate on longitudinal data in AARC (with at least one wave of data collection of AARC before the occurrence of the life events) to disentangle the potentially complex mechanisms in its association with life events.

Lastly, future research may include further constructs and research questions to (a) investigate for whom and under which circumstances life events affect AARC, and (b) investigate mediating variables and potential consequences of the association between life events and AARC. Regarding the former, aspects of coping, personality, control, social and instrumental support, upward and downward social comparisons, but also characteristics of the life event itself could be of relevance (17, 50). Regarding the latter, a high awareness of age-related losses could for example function as a mediator in the association between life events in the health domain and mental health (2).

## Conclusion

When investigating AARC in the context of life events in an adult lifespan sample, we found evidence that the cumulative experience of life events was related to a higher awareness of both age-related gains and age-related losses. The experience of life events in the family domain featured prominently in the awareness of age-related gains, whereas the experience of life events in the health domain featured more strongly in the awareness of age-related losses. The experience of life events and the associated change may impact how individuals perceive their aging process, may challenge the subjective experience of age-related losses, but may also foster the subjective experience of age-related gains.

## Data availability statement

The raw data supporting the conclusions of this article will be made available by the authors, without undue reservation.

## Ethics statement

This study was a secondary analysis of anonymized data, and therefore an ethics approval was not required. Detailed information on ethical clearance related to the German Socio-Economic Panel (SOEP) can be found on their website (https://www.diw.de/soep).

## Author contributions

FR: conception and design of the work, analyses, and writing. SS: contribution to conception and design of the work and analyses. MD, DG, RK, OS, and H-WW: conceptual contribution, acquisition and interpretation of data, and revising the work. All authors contributed to the article and approved the submitted version.

## Conflict of interest

The authors declare that the research was conducted in the absence of any commercial or financial relationships that could be construed as a potential conflict of interest.

## Publisher's note

All claims expressed in this article are solely those of the authors and do not necessarily represent those of their affiliated organizations, or those of the publisher, the editors and the reviewers. Any product that may be evaluated in this article, or claim that may be made by its manufacturer, is not guaranteed or endorsed by the publisher.

## Footnotes

^1^For example, based on age distributions of the current data, losing one's mother (life event *death of mother*) was most common between the ages of 50 and 65 years (= interquartile range of the age of all participants reporting the life event) and would be rated as age-corresponding when occurring within this interval. It would be rated as relatively early when occurring before the age of 50 and as relatively late when occurring after the age of 65.
